# Usefulness of Non-Contact Infrared Thermometer for Early Neonatal Period Using Bland-Altman Analysis

**Published:** 2020-02

**Authors:** Saori SUGAWARA, George IMATAKA, Shigemi YOSHIHARA

**Affiliations:** Department of Pediatrics, Dokkyo Medical University, Tochigi, Japan

## Dear Editor-in-Chief

It is impossible to measure central body temperature in a living body. Therefore, usually, body temperature is measured in the oral cavity (sublingual temperature), anus (rectal temperature), ear (tympanic temperature), armpit (axillary temperature) and others. In most cases, oral and rectal temperatures are usually used. On the other hand, in Japan in order to respect hygiene measures, body temperature is often measured in the axilla (1).

There are three types of thermometers that are used for temperature readings in Japan: mercury thermometer, ear thermometer, and electronic thermometer. Mercury thermometer that have been used for a long time is the most accurate (2). However, the measurement can take about 5 minutes. In addition, the use of mercury is on decline due to environmental pollution. Ear thermometer measure the body temperature using infrared rays emitted from the eardrum. The measurement time of this thermometer is a few seconds. However, measurement errors are likely to occur in newborns and infants because the external auditory canal is narrow and the amount of exudate is large. At the Japanese pediatric hospital electronic thermometers are often used for newborns and infants.

In recent years, in addition to these three thermometers, non-contact infrared thermometers have begun to spread mainly in East Asia such as Japan and Korea. The new thermometer has the shortest measurement time (less than 1 second). However, because this type of thermometer is non-contact type, measurement errors may occur depending on the environmental temperature and humidity. Furthermore, there are few basic data in early postnatal neonates with a lot of vernix on the skin (3–7).

Therefore, we examined the usefulness of a non-contact infrared thermometer for normal neonates in the neonatal room of obstetrics in Koike Ladies Clinic in Japan. This study was approved by the hospital and informed consent was taken from the parents of participants before the study. Eighteen healthy early neonates from 0 to 6 days after birth were enrolled in this study. The neonatal gestational age was 38.1–41.0 weeks (mean±SD=39.5weeks±0.7 days, median [IQR]=39.5 weeks). The birth weight was 2,650 g to 3,306 g, (mean±SD= 2,952.7±172. g, median [IQR]= 2,988.5 g). The body temperature was measured at the center of the forehead and the axilla. Non-contact infrared thermometer (Thermo Finder Pro FS-300 manufactured by HuBDIC- Global CO., LTD, Korea) was used at the distance of 2 cm from the center of forehead.

A contact-type electronic thermometer (Terumo Thermometer C205 manufactured by Terumo Corporation, Japan) was used for the axilla. Both non-contact and contact thermometers were measured at the same time, 3 times a day, for a total of 67 times. All of newborns were examined in the supine position on a newborn cot. The room temperature was 23 to 26 degrees, and the humidity was 40 to 60%. Comparison of measurements using both thermometers was statistically analyzed using Bland-Altman analysis (8).

**Fig. 1:**
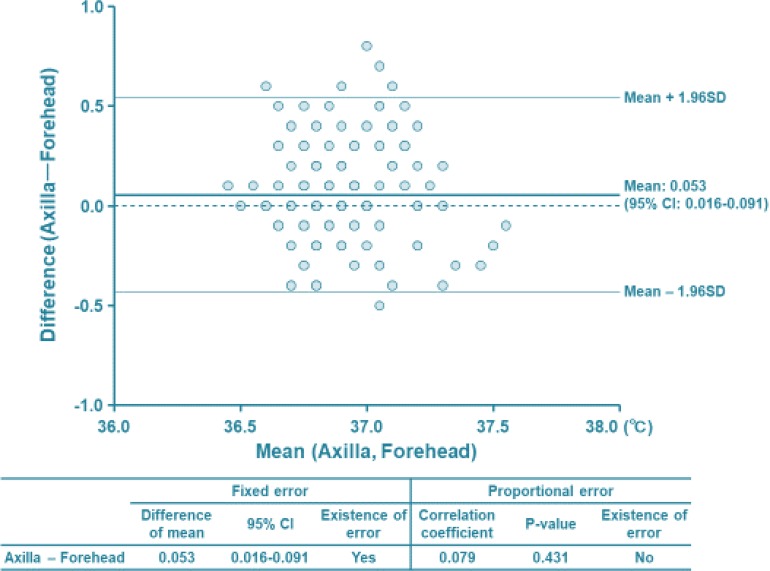
Bland-Altman analysis of our study relation between contact type of electrical thermometer at the axilla and non-contact type of infrared type of thermometer at the center of forehead in early neonate

The software used for data analysis was SPSS version 22.0 (IBM Japan, Ltd., Tokyo, Japan). The measurement results (contact and non-contact) were analyzed using Bland-Altman plots (Fig. 1).

The X-axis in the figure is the average temperature measured at the axilla and forehead. The Y-axis was plotted by subtracting the forehead temperature from the axilla temperature. In order to confirm the fixed error, the average value obtained by subtracting the forehead value from the axilla was shown as “Mean” on the horizontal line in the figure. Analysis of mean body temperature showed that the axilla body temperature was 0.053 °C higher than the forehead temperature. The 95% confidence interval was 0.016–0.091. Since this confidence interval does not cross 0, it was judged that the analysis was a significant fixed error. In addition, since the correlation coefficient was 0.079 and the *P* value was 0.431, there was no proportional error.

Temperature measurements in the axilla were significantly 0.053 °C higher than forehead. The 0.053 °C difference is clinically acceptable. So, both contact-type electronic thermometer and non-contact-type infrared thermometer in newborns have no clinical problems in measuring temperature.

One of the difficulties we encountered when measuring the body temperature was horizontal infection mainly due to MRSA and *Pseudomonas aeruginosa* due to skin contact. Furthermore, it was difficult to keep a newborn baby with a long sleep time resting. Non-contact thermometers did not directly contact the skin, so there was no risk of skin infection.

In conclusion, the non-contact infrared thermometer using in the neonatal room of obstetrics was not only practical but also very useful. This method was hygienic, had a short measurement time and did not disturb the quality of daily life of the newborns.
